# Rare Metallic Allergy Reaction Presentation to Cholecystectomy Surgical Clip

**DOI:** 10.7759/cureus.32934

**Published:** 2022-12-25

**Authors:** Kaveh Mozafari, Shanynn Santos, Shirali Ohri, Manish Prajwal Mane Manohar, Frederick Tiesenga

**Affiliations:** 1 Surgery, West Suburban Medical Centre, Oak Park, USA; 2 Medicine, St. George's University School of Medicine, St. George, GRD; 3 Surgery, West Suburban Medical Center, Oak Park, USA; 4 Surgery, Aureus University School of Medicine, Oranjestad, ABW; 5 Surgery, Avalon University School of Medicine, Willemstad, CUW; 6 General Surgery, West Suburban Medical Center, Chicago, USA

**Keywords:** surgical clip, minimally invasive surgical procedures, hypersensitivity reaction, conventional laparoscopic cholecystectomy, surgical staples, nickel allergy, titanium allergy, metal allergies, intra-operative cholangiogram, allergen-producing metal composites

## Abstract

Metal allergies have been a growing concern in the general population over the past several decades. Laparoscopic cholecystectomy is the standard treatment for gallbladder diseases such as cholelithiasis and cholecystitis, during which surgical clips composed of metals such as nickel or titanium are often used to clamp the arteries and ducts. These metals are documented to produce hypersensitivity reactions. Here, we present the case of a 53-year-old male patient who successfully underwent laparoscopic cholecystectomy and two weeks later reported constant right upper quadrant pain accompanied by nausea that was exacerbated by exercise and food. After several months of severe interference with the patient’s lifestyle, we removed the surgical clips after ruling out all possible organic causes of the pain. A total of six surgical clips were removed during surgery, and the patient reported a substantial resolution of symptoms post-operation. Post-cholecystectomy syndrome (PCS), allergy to the metallic surgical clips, and migration or improper clip placement during surgery were all considered possible causes for the pain. Still, the clinical presentation and laboratory studies pulled focus toward metallic surgical clip allergy as the most plausible cause for the presenting symptoms. The metallic haptens released by the surgical clips activate the innate and adaptive immune response cascades and pre-sensitize the CD8 and B cells to the metallic allergens. With reexposure, these pre-sensitized CD8 and B cells trigger a hypersensitivity reaction. Standardizing allergy tests as part of the pre-operation checklist can prove to be an inexpensive way to eliminate such post-surgical complications. Furthermore, alternatives like absorbable sutures or even different hypoallergenic metal or plastic clips can be considered viable options to replace nickel or titanium-made surgical clips during surgery.

## Introduction

Metal allergies have been a growing concern in the general population over the past several decades [1]. These are most commonly associated with nickel, chromium, cobalt, and titanium, which are often found in jewelry, coins, implant clips or devices, and dental materials. Metal allergies are considered a type IV hypersensitivity and are seen in 10-15% of the general population, more often in women than in men [2,3]. It presents as a type of contact dermatitis but can present with other non-specific symptoms such as pain, swelling, lichen plants, dyshidrotic eczema, and burning mouth syndrome are some syndromes that can present because of nickel allergy [4]. Physical findings during the initial stage of the reaction can include induration, bullae, erythema, bullae, vesicles, scaling plaques, and edema. Determinations during the later reactions include hyperkeratosis, dryness, scaling, pruritus, lichenification, fissuring, and hyperpigmentation [5].

In this case, a laparoscopic cholecystectomy was performed, which is the standard treatment for gallbladder diseases such as cholelithiasis and cholecystitis. Usually, this surgery will relieve symptoms caused by the disease or injury; however, this is not always the case. During these procedures, surgical clips made from common metals such as nickel or titanium are often used to clamp the arteries and ducts. This is where a problem can present itself [1,2]. Diverse metals are known to elicit an allergic reaction, yet nickel is the most recurring cause of metal allergy [2]. It is estimated that close to 17% of females and 3% of males are allergic to nickel [3].

## Case presentation

A 53-year-old male patient experienced constant right upper quadrant pain accompanied by nausea two weeks after his uncomplicated laparoscopic cholecystectomy surgery in 2019 to treat complications caused by cholelithiasis. He came into a surgical outpatient clinic in 2022 and revealed the pain was amplified when he positioned himself in specific positions during exercise and when he ate particular foods. He mentioned he was sensitive to casein, a protein found in milk, and had experimented to see if certain foods made him feel better or worse. As per the patient, the consumption of sweet edibles exacerbated the pain, while cooked rice alleviated the discomfort. Additionally, ingestion of gluten-free products did not improve the patient's symptoms. Due to experiencing pain and nausea two weeks after his laparoscopic cholecystectomy, he was evaluated for a metal allergy as it was suspected that the six metallic surgical clips used during his surgery in 2019 were the most probable cause of his symptoms. A metal sensitivity test confirmed that he was sensitive to nickel. He also reported a loss of appetite due to the pain and nausea, which led him to lose forty-four pounds between his surgery in 2019 and his outpatient clinic visit in 2022.

As the patient lost 44 pounds after the surgery in 2019, caused by a lot of pain and the diminishment of appetite pertained from the pain, she underwent another uneventful surgery in 2022 to remove the clips in the hope of eliminating the adverse effects caused by allergic nickel reactions. The second surgery in 2022 removed the six metallic surgical clips: two were from the cystic duct, two were from the cystic artery, and two were from the liver bed. No scars were noted in the area of the initial laparoscopic cholecystectomy surgery in 2019. The metallic surgical clips were found to be composed of nickel and titanium.

A week after the nickel and titanium surgical clips were removed, the patient revealed that the pain had decreased in intensity and had become dull and more diffuse. He described feeling just a slight pressure in the right upper quadrant area compared to the constant pain he felt prior to removing the metallic surgical clips. The patient also disclosed that he was not experiencing a loss of appetite anymore due to the reduction of pain and nausea.

**Figure 1:**
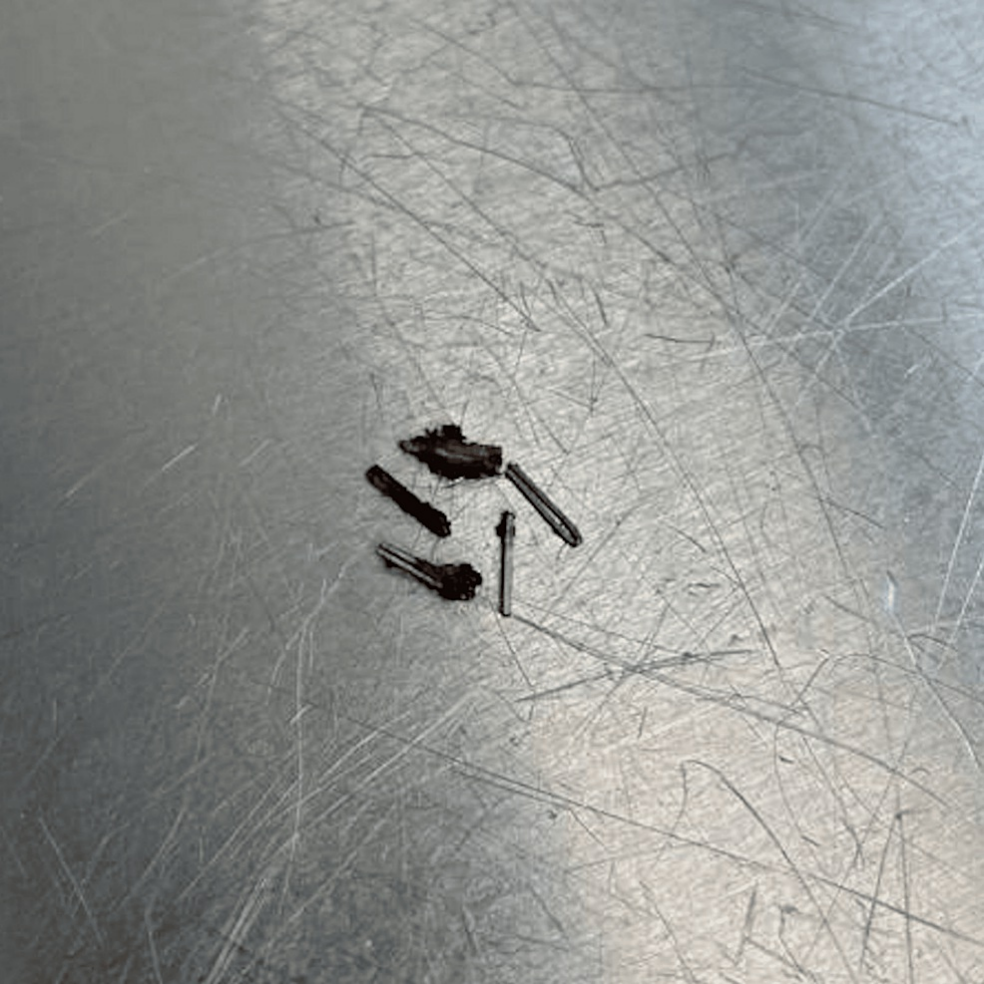
The extracted surgical clips were found to be composed of nickel and titanium.

As the patient’s right upper quadrant pain and nausea significantly declined after the successful removal of the six metallic surgical clips, it can be confirmed that the symptoms the patient experienced after his laparoscopic cholecystectomy surgery in 2019 were in fact due to an allergic reaction to the nickel in the surgical clips (Figure 1).

## Discussion

Right upper quadrant pain after a cholecystectomy can be due to several causes, such as postcholecystectomy syndrome (PCS), allergy to the metallic surgical clips, and migration or improper clip placement during surgery. Only after ruling out all other organic causes of the right upper quadrant pain in a post-cholecystectomy patient must one consider the pain to be a complication of cholecystectomy.

Allergic reaction to the clips

Nickel and zirconium are a few of the fundamental elements found in the metallic surgical clips used in everyday practice [4]. Both of the metals are documented to produce hypersensitivity reactions in various patients, which can be one of the causes of post-cholecystectomy pain. The haptens released by the metals make the skin cells produce cytokines like IL-1b and TNF-a, which initiate an innate immune response by activating the antigen-presenting cells [6]. The activated APCs, in turn, activate the adaptive immune response cascade, where the activated CD4 T cells sensitize the CD8 cells and B cells to the allergen [7]. These pre-sensitized CD8 and B cells trigger a hypersensitivity reaction with reexposure to the metals [6]. Therefore, standardizing metallic allergy tests as part of the preoperative testing routine can reduce postoperative complications like hypersensitivity reactions and help avoid unnecessary surgical interventions to remove the clips. Furthermore, alternatives like absorbable surgical sutures can be considered to replace the metal clips in patients who test positive for metallic allergies. Multifarious metals are known to provoke an allergic reaction, yet nickel is the most recurring cause of metal allergy [2]. It is estimated that 17% of females and 3% of males are allergic to nickel [3]. The best way to avoid postoperative issues is to routinely ask patients before surgery if they have any known metal hypersensitivity, including problems with cosmetic jewelry [8]. A preoperative allergy test is cost-effective and non-invasive compared to a postoperative surgical clip extraction, and it prevents the patient from further surgical complications. However, there are some limitations associated with the preoperative metallic allergy tests, as not all institutions routinely perform metallic clip allergy testing due to rare occurrences. Polymeric locking clips can also be envisioned as an alternative option [9], yet the cost limitation must be considered when compared to metallic clips.

Postcholecystectomy syndrome

PCS refers to the right upper quadrant pain experienced by the patient after the cholecystectomy surgery with several other gastrointestinal symptoms similar to what the patient experienced before the surgery [10]. The symptoms can include abdominal pain, nausea, vomiting, diarrhea, heartburn, and intolerance to a high-fat diet. Based on the source of the symptoms, PCS may be further categorized into biliary and non-biliary manifestations. Biliary causes can be due to incomplete surgery with retained calculi, biliary strictures, complications of the sphincter of Oddi, and biliary stenosis [11]. Other organic causes like pancreatic, esophageal, and gastroenteric pathologies may be considered under the non-biliary manifestations of the PCS [10]. It is also important to note that improper placement of the clips during surgery or migration of the clips can cause friction. This can result in inflammation of nearby local structures like the biliary tree, leading to long-term cholangitis, which can cause right upper quadrant pain unexplained by any other organic cause [12]. Additionally, non-GI causes like psychiatric and local neurological pathologies can mimic PCS symptoms [10].

Surgical intervention to remove the clips must be considered if the patient’s lifestyle is severely affected, like in the case of this patient, whose pain was so genuinely concerning that it started hindering his lifestyle. Hence, the surgical clips were removed empirically when it was confirmed that any other organic cause could not explain the pain. Removing the clips not only helped us rule out the possibility of iatrogenic misplacement and migration of the clips as the origin of pain but also resolved the issue of any possible hypersensitivity against the clips. Therefore, minimally invasive surgery should be considered a viable and effective management source to provide the patient with a better lifestyle.

## Conclusions

There are many possible complications post-surgery, including delayed hypersensitivities to nickel or other metal alloys. Increasing awareness of sensitivities and questioning the patient before procedures can help eliminate some of these complications so that they are avoided altogether. Currently, some other viable options can be used in place of surgical clips, such as absorbable sutures or different metal options if a specific allergy is known. Polymeric locking clips can also serve as an alternative option for patients with a high degree of suspicion for a possible metallic allergy. As allergies to metals continue to increase, awareness and prevention will become key factors in any decisions before surgical intervention.
